# Social determinants of malnutrition in Chilean children aged up to five

**DOI:** 10.1186/s12889-021-12455-4

**Published:** 2022-01-07

**Authors:** Sandra Alvear-Vega, Héctor Vargas-Garrido

**Affiliations:** 1 Faculty of Economics, University of Talca, Talca, Chile; 2 Faculty of Psychology, University of Talca, Talca, Chile

**Keywords:** Social determinants, Undernutrition, Overnutrition, Children, Under-five-years

## Abstract

**Background:**

This study aimed to ascertain the Social Determinants (SDs) of malnutrition (over and undernutrition) of Chilean children aged up to five.

**Methods:**

The study was carried out using a sample of children from zero to five years old (*n* = 1,270,485; 52.2% female) from the National Socioeconomic Characterization Survey (CASEN) 2017. A multinomial logistic regression model was used, where the “child nutritional status” outcome variable assumed three possible values: normal nutrition, overnutrition, and undernutrition, while taking those variables reported in previous literature as independent variables.

**Results:**

The model, by default, set normal nutrition as the reference group, Count R2 = 0.81. Results show a higher likelihood of both overnutrition and undernutrition among male children from the lowest quintiles, with native ethnic backgrounds, reporting health problems, having public health insurance, and who attend kindergarten. Additionally, higher probabilities of undernutrition in younger than two and living in the north of the country, while overnutrition is more likely in the south.

**Conclusions:**

Socioeconomic variables are fundamentally related to both over and undernutrition; the current single schema program to prevent malnutrition should consider SDs such as ethnicity and geographical location, among others; moreover, successful nutritional programs—which focused on the lowest quintiles, need to be expanded to other vulnerable groups and pay more attention to overnutrition.

## Background

To understand the causes of diseases and unhealthy behavior, the literature needs to know the social conditions that underlie people’s health, called “the social causes of disease” [1]. Knowledge of social determinants (SDs) can contribute to implementing comprehensive social programs to address the conditions that generate disadvantages to people’s health [2]. For example, unfavorable socio-economic conditions are one SD that show greater consistency and persistence over time [3–5]. Furthermore, age [6], gender [3], and demographic and ethnic conditions [2, 7] are usually relevant, among others.

### Social determinants of malnutrition in children under five

Malnutrition in children under five—either under or overnutrition—is a matter that requires serious attention because it increases the risk of illness or death, worsens comorbidities, and negatively impacts physical, intellectual, and psychomotor development [2, 6, 8, 9]. Moreover, children with undernutrition are more likely to live in a context of poverty and inequality in their adulthood, which perpetuates the risk of undernutrition for future generations, creating a harmful cycle that is difficult to eradicate [4]. On the other hand, those with overnutrition have detrimental academic performance and, in addition, mental health problems linked to low self-esteem. Likewise, child malnutrition implies losing a healthy lifetime [10], increasing health costs [9], and negatively affecting future human capital [11].

Malnutrition in children under five can result either from a deficit or excess in energy intake, both within the same population, or at different times in a child’s lifespan. For example, in Latin America and the Caribbean, children’s obesity has increased dramatically, while undernutrition continues to be a challenge [8]. This so-called “double burden” of child malnutrition is related to the nutritional transition process, a public health problem that deserves greater attention from an epidemiological point of view [12]. It should be noted that, although both types of malnutrition are associated with different energy intakes and reveal certain differences in their SDs [5, 8], they should be approached from a comprehensive perspective [13].

To classify the underlying and immediate SDs of malnutrition, Harris and Nisbett [14] identified three overall factors: a) material, human, and social sources; b) structural (society, markets, law, political); and c) ideas and beliefs. Previous studies have found conditions that could encompass this classification. For example, regarding undernutrition of children from zero to five, some key SDs are parents’ educational level—mainly the mother, access to drinking water, and sanitation facilities [4, 6, 15, 16]. In this vein, the mother figure is critical, as a greater vulnerability is found in children of single mothers with a low income, chronic diseases, and lacking health insurance. Combined, these conditions mean it is less likely the mother could stay with the child or provide a substitute caregiver [5]. On the contrary, maternal higher body mass index and higher educational level are associated with a lower likelihood of child undernutrition [6]. Unsurprisingly, underlying those conditions is household income, which is a significant determinant of childhood undernutrition [3, 4, 6, 7, 15]. Similarly, the community’s role is relevant through social support and knowledge sharing concerning access to social protection programs and enabling collective demand for better public services [4, 5]. In addition, Hossain et al. [3] revealed that the odds of undernutrition decrease in children breastfed for periods of longer than six months or with complementary feeding. In terms of gender, there appear to be geographical differences. For example, in Nepal, a lower risk of undernutrition was found in male children [3], while the opposite is true in Indonesia [17]. Additionally, higher probabilities of undernutrition have been observed in some countries’ geographical areas, among ethnic communities, and in less urban locations [7, 15].

On the other hand, overnutrition (i.e., overweight and obese) is related to increasing urbanization [18, 19], new ways of both transportation and food commercialization, and the development and use of technologies that increase physical inactivity and unhealthy food consumption [20–22]. Considering the transition from greater undernutrition to greater overnutrition, several observed mediating variables are the change from rural to urban, fewer people and children in the household, and access to prenatal care for mothers [18]. Overnutrition is a rising problem worldwide [23–25], which for under-fives is related to babies younger than 36 months and overweight parents [13, 24], with a higher prevalence in some geographical areas [26]. However, there are other SDs related to overnutrition in under-fives that have less consistency in previous research. For example, higher odds of being overweight have been reported when the mother has a higher education level and when the child belongs to families with higher incomes than those with lower incomes [8, 24]. However, other studies reveal a relationship between overnutrition with lower household income, neighborhood insecurity [27] and lower parental education [28].

### Malnutrition in Chilean children up to five

Chile has undergone a rapid socio-economic and nutritional transition, leading to a co-existence of the two extremes of child malnutrition, although with lower undernutrition and greater overnutrition (overweight and obese) [29]. Consequently, some researchers have claimed that the “double burden” of child malnutrition would not be present in Chile because of the low rates of undernutrition. They also emphasize that the country’s main problem would be overnutrition [30]. Nevertheless, the authorities recently highlighted the topic of undernutrition because it continues to be a public health problem that requires attention [31]; among Chilean children under five years old, the undernutrition rate is 1.8%, while 9.3% regarding overnutrition [32]. However, due to increases with age [33], it reached a worrying 54% in the case of primary school children in 2020 [34].

In Chile, measures in children under six indicate that males are more likely to be severely obese, while females are more likely to be overweight and of normal weight [34]. Similarly, male children have a slightly higher percentage when undernutrition categories are grouped (“undernourished” and “underweight”). Overnutrition arises mainly between three and five years of age [10, 35]. Likewise, previous works have evidenced that native ethnicity becomes a significant SD of undernutrition in children under five in Latin America [36] and Chile [37]. In this vein, research on indigenous Chilean children between six and eight years old has observed a higher prevalence of obesity [38].

Moreoever, Pavez-Soto et al. [39] highlighted the challenge of studying migrant children’s nutritional conditions, stating that the migrant population in Chile comes mainly from Latin American countries with similar sociocultural conditions. Thus, it would be expected to find high levels of overnutrition, as it is a characteristic seen in this region [8]. Similarly, recent works have discovered that migrant children in Chile present a higher biopsychosocial risk, lower access to social programs, and a higher proportion of multidimensional poverty [40], all factors associated with a higher probability of undernutrition [3, 4, 6, 7, 15]. Thus, all the variables mentioned above will be considered in this study.

In Chile, it is worth noting that there is a National Supplementary Feeding Program (PNAC) aimed mainly at families with public health insurance. The PNAC has a basic, single, and identical scheme for all children under six years old (only differentiating by age) but considers a reinforcement for undernourished risk [41].

Therefore, bearing in mind the relevance of malnutrition in Chile, which requires awareness of its multidimensional causes, the present study aims to identify and describe the SDs related to malnutrition in children up to five. For such purposes, the data provided by the Casen 2017 survey will be considered [42]. A multinomial logistic regression will be performed. This probabilistic model is used when the predicted outcome variable is nominal and has more than two categories—without a given rank or order. The model can be used with any number of independent variables, either categorical or continuous. Such methodology has been used previously in studies regarding the nutritional status of children and adults [43, 44].

## Material and methods

### Source of information

The data used in this study are from the National Socioeconomic Characterization Survey, CASEN 2017 [42], conducted by the Surveys and Longitudinal Studies Center of the Catholic University of Chile (PUC) for the Ministry of Social Development and Family (MINDES). The sample units of the CASEN 2017 survey are dwellings selected in a probabilistic, stratified, and multistage manner. Inside dwellings, all family units are identified (households). Interviewers applied a paper-and-pencil survey in a face-to-face procedure, interviewing the head of the family unit or an adult member in their absence. Data consisted of a sample of 26,249 households with children under five years old, which, using the expansion factor (EXPR), reached 1,270,485 children. The EXPR allows us to obtain the results of persons and family units expanded at the national and regional levels, as well as by area (urban and rural), being representative of the country’s overall population [42]. All interviewees participated voluntarily, and anonymity is ensured under Law 17.374. CASEN 2017 is used as a secondary data source [45].

### Outcome variable: undernutrition, normal nutrition, and overnutrition

CASEN 2017 asks interviewees what the child’s nutritional status is. Based on medical check-up reports, response options are 1. Undernourished or at risk of undernourishment; 2. Normal; 3. Overweight; 4. Obese. In this study, responses were grouped as undernutrition (1), normal (2), and overnutrition (3 and 4).

### Independent variables

Independent variables were extracted from the previous findings as long as they were present in CASEN 2017. CASEN has seven modules (registration-residents, education, work, income, health, identities-networks-participation, and housing-environment), from which three types of dimensions grouped the variables of interest in this research. First is the demographic dimension (including gender, age, nationality, ethnicity, urban or rural area, and geographic location). Second is the social dimension (comprising household income, educational level, type of health insurance, and child participation in the supplementary feeding program). Finally, the third dimension grouped the child’s personal features (health problems, health checkup, staying at home, and kindergarten attendance). All independent variables are dichotomous: 1 (belongs to the category) and 0 (does not) (see Table 1). It is worth noting that the nationality, belonging to a native ethnic group, household income, and household educational level variables were considered based on the survey respondents’ information.

**Table 1:** Description of the control variables, extracted from CASEN 2017

Variable name	CASEN 2017 question	Codification
Gender	Male or female?	Male = 1 Female = 0
Age ≤ 2 years	How old is s/he?	Inside the range = 1 Outside the range = 0
Age 2 ≤ 5 years	How old is s/he?	Inside the range = 1 Outside the range = 0
Nationality	What is your nationality?	Chilean = 1 Others = 0
Household educational level	What was the highest educational level reached?	Primary or secondary school =1 Technical or university = 0
Native ethnic groups	Indigenous peoples. Do you belong to or are you a descendant of any of them?	Yes = 1 No = 0
At home most of the time	Where does the child spend the most number of hours?	Home = 1 Other = 0
Kindergarten attendance	Currently, does the child attend any educational institution, kindergarten, nursery school, or other non-conventional kindergarten program?	Yes = 1 No = 0
Type of health insurance	Which health insurance system do you belong to?	Public = 1 Private = 0
Health problems	In the last three months, did s/he have any health problem, illness, or accident?	Yes = 1 No = 0
Well-child checkup	What type of checkup did s/he undergo in the last three months? Well-child checkup.	Yes = 1 No = 0
State supplementary feeding program	For children aged 0 to 6 years, in the last three months, did s/he receive or withdraw, free of charge, food from the consultation room or hospital?	Yes = 1 No = 0
Urban/rural residence	The interviewer registered this according to the survey’s design.	Urban = 1 Rural = 0
Great North Zone (1)	Grouped by CASEN. Zone 1 includes regions formerly numbered as 1, 2, and 15.	Inside the range = 1 Outside the range = 0
Little North Zone (2)	Grouped by CASEN. Zone 2 includes regions formerly numbered as 3 and 4.	Inside the range = 1 Outside the range = 0
Central Zone (3)	Grouped by CASEN. Zone 3 includes regions formerly numbered as 5, 6, 7, 13, and 16.	Inside the range = 1 Outside the range = 0
South Zone (4)	Grouped by CASEN. Zone 4 includes regions formerly numbered as 8, 9, 10, and 14.	Inside the range = 1 Outside the range = 0
Austral Zone (5)	Grouped by CASEN. Zone 5 includes regions formerly numbered as 11 and 12.	Inside the range = 1 Outside the range = 0
Quintile I	Built and delivered by CASEN.	Inside the range = 1 Outside the range = 0
Quintile II	Built and delivered by CASEN.	Inside the range = 1 Outside the range = 0
Quintile III	Built and delivered by CASEN.	Inside the range = 1 Outside the range = 0
Quintile IV	Built and delivered by CASEN.	Inside the range = 1 Outside the range = 0
Quintile V	Built and delivered by CASEN.	Inside the range = 1 Outside the range = 0

### Plan of analysis

A multinomial logistic model was estimated to establish the relationship between children up to five’s nutritional status and the independent variables. For each of the interactions, average marginal effects, likelihood, and relative risk were calculated. A statistical analysis was performed with Stata14 software. A set of coefficients, namely *β*^*1*^, *β*^*2*^, and *β*^*3*^, representing normal nutrition, overnutrition, and undernutrition, respectively, were estimated, corresponding to:

$$\mathit{\Pr}\left(y\right.=\left.1\right)=\frac{e^{x{\beta}^{(1)}}}{e^{x{\beta}^{(1)}}+{e}^{x{\beta}^{(2)}}+{e}^{x{\beta}^{(3)}}}$$

$$\mathit{\Pr}\left(y\right.=\left.2\right)=\frac{e^{x{\beta}^{(2)}}}{e^{x{\beta}^{(1)}}+{e}^{x{\beta}^{(2)}}+{e}^{x{\beta}^{(3)}}}$$

$$\mathit{\Pr}\left(y\right.=\left.3\right)=\frac{e^{x{\beta}^{(3)}}}{e^{x{\beta}^{(1)}}+{e}^{x{\beta}^{(2)}}+{e}^{x{\beta}^{(3)}}}$$

Relative likelihood of y = 2 to base result is

$$\frac{\mathit{\Pr}\left(y=2\right)}{\mathit{\Pr}\left(y=1\right)}={e}^{x{\beta}^{(2)}}$$

The relative risk ratio for a one-unit increase/decrease in X_i_ is:

$$\frac{e^{\beta_1^{(2)}x1+\dots +{\beta}_i^{(2)}\left( xi+1\right)+\dots +{\beta}_k^{(2)} xk}}{e^{\beta_1^{(2)}x1+\dots +{\beta}_i^{(2)} xi+\dots +{\beta}_k^{(2)} xk}}={e}^{\beta_i^{(2)}}$$

## Results

### Goodness of fit

The estimation of the model is based on 1,270,485 observations. The log-likelihood value is −706,371.45, *χ*^2^ (40) = 36,668.99, *p* < 0.0001, thus rejecting the null hypothesis that all parameters included in the model are equal to zero. Likewise, goodness of fit, Count R2 = 0.81, indicates the model has good explanatory power. The independent variables explain the outcome variable between 2.8% (Cox-Snell) and 4.1% (Nagelkerke) (see Table 2).

**Table 2:** The goodness of fit statistics

Number of observations	1.270.485
Iterations	-706371.45
LR Chi-square (40)	36668.99
Prob > chi2	0.000
Count R2	0.812
McFadden’s R2	0.025
Cox-Snell	0.028
Nagelkerke	0.041

The model, by default, set normal nutrition as the reference group and estimated a model for overnutrition and a model for undernutrition, both of which were relative to normal nutrition. According to the confidence intervals for the relative risk, with 95% confidence, the relative risk ratio is proper.

### Marginal effects

Table 3 illustrates the increasing or decreasing likelihood of a child under five having normal nutrition, overnutrition, and undernutrition due to the independent variables. The marginal effects (likelihood) were slightly more significant for overnutrition compared to either undernutrition or normal nutrition.

**Table 3:** Multinomial logistic regression marginal effects

	Predict (Normal Nutrition)		Predict (Overnutrition)		Predict (Undernutrición)	
	dy/dx	[95%. Conf. Interva]	dy/dx	[95%. Conf. Interv.]	dy/dx	[95%. Conf. Interv.]
Gender	-0.0104 ***	[-0.0117; -0.0091]	0.0050***	[0.0038; 0.0063]	0.0053***	[0.0047; 0.0059]
Age ≤ 2 years	0.0191***	[0.0175; 0.0207]	-0.0302***	[-0.0317; -0,0287]	0.0111***	[0.0103; 0.0118]
Age 2 ≤ 5 years (base)	-	-	-	-	-	-
Nationality	-0.0752***	[-0.0785; -0,0720]	0.0915***	[0.0890; 0.0941]	-0.0162***	[-0.0183; -0.0141]
Household educational level	0.0443***	[0.0327; 0,0559]	0.0163***	[0.0065; 0.0261]	-0.0607***	[-0.0682; -0.0532]
Native ethnic groups	-0.0223***	[-0.0244; -0.0201]	0.0202***	[0.0182; 0.0222]	0.0020***	[0.0010; 0.0029]
At home most of the time	0.0377***	[0.0347; -0.0407]	-0.0306***	[-0.0334; -0.0279]	-0.0070***	[-0.0085; -0.0056]
Kindergarten attendance	-0.0324***	[-0.0356; -0.0292]	0.0305***	[0.0276; 0.0334]	-0.0018*	[0.0004; 0.0033]
Type of health insurance	-0.0316***	[-0.0337; -0.0295]	0.0319***	[0.0300; 0.0338]	0.0003	[-0.0013; 0.0006]
Health problems	-0.0452***	[-0.0469; -0.0435]	0.0360***	[0.0347; 0.0379]	0.0086***	[0.0080; 0.0096]
Well-child checkup	-0.0369***	[-0.0382: -0.0355]	0.0319***	[0.0306; 0.0332]	0.0049***	[0.0043; 0.0055]
State supplementary feeding program	-0.0399***	[-0.0415: -0.0384]	0.0343***	[0.0329; 0.0357]	0.0559***	[0.0049; 0.0062]
Urban/rural residence	0.0250***	[0.0229; 0.0272]	-0.0208***	[-0.0228; -0.0188]	-0.0042***	[-0.0051; -0.0032]
Great North Zone (1)	0.0306***	[0.0281; 0.0331]	-0.0402***	[-0.0422; -0.0378]	0.0093***	[0.0080; 0.0106]
Little North Zone (2)	0.0294***	[0.0269; 0.0320]	-0.0312***	[-0.0335; -0.0289]	0.0017**	[0.0005; 0.0029]
Central Zone (3) (base)	-	-	-	-	-	-
South Zone (4)	-0.0006	[-0.0023; 0.0011]	0.0039***	[0.0023; 0.0055]	-0.0033***	[-0.0040; -0.0025]
Austral Zone (5)	-0.0294***	[-0.0355; -0.0233]	0.0192***	[0.0136; 0.0248]	0.0102***	[0.0071; 0.0132]
Quintile I	-0.0747***	[-0.0782; -0.0713]	0.0510***	[0.0478; 0.0541]	0.0237***	[0.0218; 0.0256]
Quintile II	-0.0936***	[-0.0971; -0.0900]	0.0623***	[0.0591; 0.0656]	0.0312***	[0.0291; 0.0332]
Quintile III	-0.0811***	[-0.0848; -0.0775]	0.0604***	[0.0570; 0.0638]	0.0207***	[0.0187; 0.0226]
Quintile IV	-0.0633***	[-0.0670; -0.0596]	0.0548***	[0.0513; 0,0583]	0.0084***	[0.0066; 0.1023]
Quintile V (base)	-	-	-	-	-	-

*p < .05; **p < .01; ***p < .001

The variables that increased the likelihood of normal nutrition are household educational level (4.4%), at home most of the time (3.7%), and Great North Zone (3.0%), while variables that decreased this are: quintile II (−9.3%), nationality (−7.5%), and health problems (−4.5%) (see Table 3).

Likewise, variables that increase the likelihood of overnutrition are nationality (9.1%), quintile II (6.2%), health problems (3.6%), participations in the state supplementary feeding program (3.4%), and type of health insurance (3.1%). On the contrary, variables that decreased this likelihood are Great North Zone (−4.0%), at home most of the time (−3.0%), and age ≤ 2 years (3.0%) (see Table 3).

Regarding undernutrition, variables that increased the probabilities are participation in the state supplementary feeding program (5.5%), quintile II (3.1%), and age ≤ 2 years (1.1%), while those that decreased it are: household educational level (−6.0%) and nationality (−1.6%) (see Table 3).

### Relative risk

From the first analysis level based on relative risk, it is possible to infer the likelihood that a Chilean child up to five years of age shows over or undernutrition regarding a normal nutritional state (see Table 4).

**Table 4:** Multinomial logistic model relative risk

	Predict (Nutrition)				
	Normal	Overnutrition		Undernutrition	
	Base Outcome	Relative Risk RRR	95% Conf. Interval	Relative Risk RRR	95% Conf. Interval
Gender		1.04***	[1.0380; 1.0584]	1.21***	[1.1904; 1.2388]
Age ≤ 2 years		0.79***	[0.7862; 0.8055]	1.41***	[1.3811; 1.4510]
Age 2 ≤ 5 years (base)		-	-	-	-
Nationality		2.73***	[2.6199; 2.8575]	0.70***	[0.6667; 0.7355]
Household educational level		1.06	[0.9757; 1.1591]	0.30***	[0.2813; 0.3383]
Native ethnic groups		1.17***	[1.1534; 1.1879]	1.09***	[1.0653; 1.1323]
At home most of the time		0.77***	[0.7587; 0.7929]	0.75***	[0.7134; 0.7900]
Kindergarten attendance		1.27***	[1.2497; 1.3091]	1.10***	[1.0530; 1.1686]
Type of health insurance		1.30***	[1.2872; 1.3320]	1.02	[0.9932; 1.0627]
Health problems		1.32***	[1.3153; 1.3457]	1.38***	[1.3623; 1.4269]
Well-child checkup		1.29***	[1.2849; 1.3115]	1.23***	[1.2131; 1.2651]
State supplementary feeding program		1.33***	[1.3175; 1.3490]	1.27***	[1.2452; 1.3071]
Urban/rural residence		0.84***	[0.8362; 0.8608]	0.84***	[0.8216; 0.8719]
Great North Zone (1)		0.70***	[0.6936; 0.72521]	1.27***	[1.2295; 1.3223]
Little North Zone (2)		0.76***	[0.7485; 0.7818]	1.02	[0.9813; 1.0638]
Central Zone (3) (Base)		-	-	-	-
South Zone (4)		1.03***	[1.0145; 1.0401]	0.89***	[0.8672; 0.9155]
Austral Zone (5)		1.17***	[1.1256; 1.2200]	1.39***	[1.2896; 1.5150]
Quintile I		1.51***	[1.4798; 1.5475]	2.17***	[2.0731; 2.2829]
Quintile II		1.65***	[1.6150; 1.6878]	2.61***	[2.4957; 2.7429]
Quintile III		1.59***	[1.5572; 1.6286]	1.97***	[1.8831; 2.0756]
Quintile IV		1.50***	[1.4679; 1.5364]	1.40***	[1.3332; 1.4786]
Quintile V (Base)		-	-	-	-
Constant		0.02***	[0.0271; 0.0331]	0.05***	[0.05062; 0.0638]

*p < .05; **p < .01; ***p < .001

There is a greater probability of over and undernutrition in male children, those who belong to native ethnic groups, those who attend kindergarten, those with health problems, and those who attend well-child checkups through the public health system (Table 4). In addition, the medical checkup and receiving complementary food variables increase the probability of over and undernutrition, compared to a normal nutritional status. Likewise, if the child’s home is in an urban area, the likelihood of over or undernutrition decreases by the same amount; 0.84 times less.

Likewise, there is a 1.04, 2.73, and 1.17 times greater probability of overnutrition if the child is male, has a Chilean background, and belongs to a native ethnic group, respectively. In parallel, if the child attends kindergarten and has health problems, they are more likely to be overnourished by 1.27 and 1.32, respectively (Table 4). In addition, if the child’s home is in the South and Austral Zone of the country, the probability of overnutrition increases.

On the other hand, there is a 1.21, 1.41, and 1.09 times greater probability of undernutrition in male children, those under two years old, and those that belong to native ethnic groups, respectively. If the child attends kindergarten and has health problems, the probability of undernutrition increases by 1.10 and 1.38, respectively. Likewise, if the child’s home is in the north zone of the country (Great North), the probability of undernutrition increases by 1.27 (see Table 4).

Moreover, as income decreases, i.e., moving down from Quintile IV to Quintile I, the probability of both under- and overnutrition among children increases compared to children in the highest income Quintile (V). However, this trend differs regarding Quintile I, which has a lower probability of over and undernutrition than Quintile II (see Table 4).

## Discussion

Using the CASEN 2017 survey, our results on the SDs of malnutrition in Chilean children up to five converge with previous findings and expand the literature’s understanding of this critical public health topic. Our discussion will follow the dimensions proposed in the methodology: demographic, social, and personal child’s features.

Regarding demographic variables (gender, age, ethnicity, migrant status, and geographic location), there is evidence that malnutrition in children differs in sex by country [3, 17, 34]. Our results are in the same direction. Boys have a lower probability of exhibiting normal nutrition than girls; consistently, male children present higher probabilities of under- and overnutrition. It should be noted that this scenario contrasts with adulthood when women have higher levels of overnutrition in Chile [33]. This interplay should search for sociocultural answers such as type of food, physical activity patterns, and gender stereotypes [4, 19, 46].

Children younger than 24 months are more likely to suffer from undernutrition in Chile. This condition has been observed elsewhere [47] and is mainly due to children who stop being breastfed becoming exposed to water, food, and environmental contamination, which occur between the first and second years of life. Likewise, we confirmed that overnutrition is more likely between three and five years old in Chile [10, 35]. Consequently, a protective factor in avoiding malnutrition—under and over—is when the child is breastfed for longer than the first six months [3, 48].

Children belonging to native ethnicities are more likely to suffer from both under- and overnutrition, as previously observed [37, 38]. However, rather than the ethnicity itself, these findings are explained by the environmental conditions and greater vulnerability of these children’s communities [36–38].

To compare migrant status, respondents’ nationality was considered, as we were interested in children’s backgrounds (every child born in Chile is of Chilean nationality, which is highly likely in the group investigated). Although there are high rates of overnutrition in Chilean children as a whole [49], our results show that children from Chilean backgrounds have a significantly higher risk of overnutrition and a lower risk of undernutrition than children of other nationalities, which would be compatible with previous findings [8, 40].

Regarding children’s geographic location, previous studies have found that several regions in other countries display a higher prevalence of malnutrition, either by deficit [7, 15] or by excess [13, 26]. In this study, children living in the north zone of Chile (Great and Little North) are less likely to be in the overnutrition category than those living in the Central Zone, while those in the Great North Zone are more likely to be in the undernutrition category than the Central Zone. Likewise, compared to the Central Zone, children living in the South and Austral Zones have a higher probability of overnutrition, and the Austral Zone also has a higher probability of undernutrition. Consequently, our results coincide with national statistics that show overnutrition increases from Chile in a north to south direction [10], which seems to be related to climate and food patterns linked to geographical characteristics. For example, there is some evidence that the South and Austral Zones’ higher levels of overnutrition are due to the low consumption of dairy products, fruits and vegetables, and a high level of sedentarism. Although this information concerns early schoolchildren [50, 51], it appears to be applicable to our findings. Similarly, South and Austral Zones exhibit a higher prevalence in native ethnic families, who are especially vulnerable to malnutrition [37, 38].

Following geographic location, children residing in urban areas are less likely to belong to the over- and undernutrition categories than children living in rural areas. International evidence consistently reports higher chances of undernutrition in less urban communities [7, 15], including Chile [38]. On the other hand, although overnutrition is greatly related to urbanization [18, 19], recent Chilean reports with children aged six to eight indicate a higher probability of both normal nutrition and overweightness in urban schools, but a higher probability of obesity in rural schools [34]. Combined, these antecedents indicate that the most severe cases of malnutrition (by deficit and excess) would be in more rural conditions. Thus, since geographic location (zones of the country and rural/urban differences) is one SD in the malnutrition of children under five in Chile, our findings suggest nutritional interventions should be localized and specific as previously indicated [38].

Second, socio-economic variables are examined. Our findings align with previous evidence and help the literature to better understand what occurs within the country regarding household income. When Quintile V is used as the basis for the analyses (the highest income group), all other quintiles exhibit higher probabilities of under- and overnutrition, steadily increasing toward the poorest quintile, Quintile I. In this regard, robust previous evidence proved that a lower household income is a predictor for undernutrition [3, 4, 6, 7, 15]. Likewise, it was recently reported that a lower family income is associated with a higher likelihood of overnutrition in Chile [27]. However, there is a change in the trend for Quintile I (the lowest income group), which shows fewer overnutrition odds than Quintiles II and III and a less severe undernutrition likelihood than Quintile II. Furthermore, previous research on Latin America has observed that the probability of overnutrition is more remarkable in families of higher incomes compared to those who are less advantaged [8, 24], which specifically occurs between Quintiles II and III compared to Quintile I in Chile. However, contrary to previous evidence, and despite having a higher household income, Quintile II has a higher probability of undernutrition compared to Quintile I. While we do not have a definitive answer for this finding, to some extent it might occur because of targeting by social programs. Access to social programs decreases children’s malnutrition [3–5], but they are sharply targeted in Chile at the least advantaged groups [52]. Therefore, this reasoning would encourage the continuation of social programs because of their outcomes, but at the same time, it warns that program targeting might lead to the neglect of other vulnerable groups.

In this same vein, considering household educational level, research consistently observed that a higher educational level is protective against children’s undernutrition [4, 6, 8]. However, it would not be related to overnutrition. The educational level of either the parents or the household in general—as SD in overnutrition—shows less consistency in previous studies (see, for example, [8, 24, 28]). This evidence points out that a higher household educational level carries a higher household income, ensuring the necessary caloric intake. Nevertheless, a higher income is not necessarily related to good food habits, nor does it guarantee a less sedentary lifestyle [19, 20, 29].

Our results ascertain that children of families with public health insurance do not have a particular risk of undernutrition compared to children from families with private insurance. Accordingly, previous findings show that health insurance is a protective factor against undernutrition in children under five years old [5]. On the other hand, our results identify a relationship in terms of belonging to the public health insurance and overnutrition groups. We must note that the lowest-income families, which match the profile of the public health system users (called FONASA), are the most vulnerable to both types of malnutrition. Chilean state action has successfully focused on preventing undernutrition with considerable positive outcomes [29]. Nonetheless, the recent nutritional transition has led to a higher prevalence of overnutrition, which has rarely been addressed and so requires more attention [20, 30].

Thirdly, we discuss the children’s personal characteristics dimension. On the one hand, under- and overnutrition goes hand-in-hand with an increased risk of becoming ill and worsening comorbidities [2, 6, 8, 9]. Our results indicate that children reporting more significant health problems and health care attendance are more likely to have either under or overnutrition, corroborating the notion that these variables are related. On the other hand, PNAC receivers are at a higher risk of malnutrition (under and over). Because PNAC’s target is children of families with public health insurance—those who belong to the lowest income quintiles, this is likely one factor that underlies our results. Similarly, there is some evidence with other beneficiary groups of complementary feeding programs in Chile (older adults) that have recognized a considerable number of users who do not withdraw or consume these products [53]. Therefore, this issue should be evaluated in the case of PNAC as well. Additionally, it has to be highlighted that the PNAC’s technical standards indicate that it is for all children “regardless of their territorial location, nationality, socio-economic status, and social security or migratory status” [41]. This criterion does not expressly include some of the findings in this work—nor in previous research [27, 37, 38]—pinpointing that variables such as geographic location, ethnicity, and socio-economic status, among others, are SDs of malnutrition risk in children up to five.

Finally, we have found that children under five who spend most of their time at home are less likely to suffer from under- and overnutrition. At home, children are primarily cared for by their mother or grandmother in developing countries [4]. Evidence shows that a protective factor for children’s nutrition is staying at home under their mother’s care or a substitute caregiver for longer [5]. Likewise, our results show that children attending kindergarten are more likely to be undernourished or overnourished. However, it should be noted that there is some evidence with schoolchildren that determines that malnutrition is not directly related to educational facilities themselves but depends mainly on children and families’ behavior [20]. Thus, the answer to the increased likelihood of malnutrition of children under five years who attend kindergarten should seek an explanation by incorporating families’ social and economic factors and whether there is adequate coordination in food intake between the home and schools.

Among the limitations, this study does not consider interactions between the control variables and their sample weighting. Results discern the likelihood of increasing or decreasing according to the increase in one unit of each variable, taking the other variables as constants into the model. However, as our results are consistent with previous ones, there is convergence validity to our findings.

## Conclusions

1.- Our findings point out that socio-economic condition is a relevant SD in under-fives’ malnutrition, both by deficit and excess. The socio-economic condition seems to underly our results based on income quintiles, having public health insurance, ethnicity, migrant status, family educational level, and access to complementary feeding programs. All of these indicate that a greater vulnerability is associated with a higher level of malnutrition.

2.- Ethnicity, gender, and geographic location are SDs of malnutrition in Chilean children under five. However, the National Complementary Feeding Program (PNAC) has a.

single schema for all children [41]. Thus, this social program should be more sensitive to the SDs observed in the present work and should also pay more attention to overnutrition.

3.- Availability of social programs decreases malnutrition [3–5]. However, social programs largely focus on the most economically disadvantaged groups in Chile [52] and prioritize undernutrition over overnutrition [20, 29, 30]. Our results support social programs’ positive outcomes but warn that targeting could neglect other social groups.

## Data Availability

The CASEN 2017 database analyzed during the current study is available from the MINDES webpage, http://observatorio.ministeriodesarrollosocial.gob.cl/encuesta-casen-2017.
